# Transcription facilitated genome-wide recruitment of topoisomerase I and DNA gyrase

**DOI:** 10.1371/journal.pgen.1006754

**Published:** 2017-05-02

**Authors:** Wareed Ahmed, Claudia Sala, Shubhada R. Hegde, Rajiv Kumar Jha, Stewart T. Cole, Valakunja Nagaraja

**Affiliations:** 1 Department of Microbiology and Cell Biology, Indian Institute of Science, Bangalore, India; 2 Ecole Polytechnique Federale de Lausanne, Global Health Institute, Station 19, Lausanne, Switzerland; 3 Jawaharlal Nehru Centre for Advanced Scientific Research, Bangalore, India; Indiana University, UNITED STATES

## Abstract

Movement of the transcription machinery along a template alters DNA topology resulting in the accumulation of supercoils in DNA. The positive supercoils generated ahead of transcribing RNA polymerase (RNAP) and the negative supercoils accumulating behind impose severe topological constraints impeding transcription process. Previous studies have implied the role of topoisomerases in the removal of torsional stress and the maintenance of template topology but the *in vivo* interaction of functionally distinct topoisomerases with heterogeneous chromosomal territories is not deciphered. Moreover, how the transcription-induced supercoils influence the genome-wide recruitment of DNA topoisomerases remains to be explored in bacteria. Using ChIP-Seq, we show the genome-wide occupancy profile of both topoisomerase I and DNA gyrase in conjunction with RNAP in *Mycobacterium tuberculosis* taking advantage of minimal topoisomerase representation in the organism. The study unveils the first *in vivo* genome-wide interaction of both the topoisomerases with the genomic regions and establishes that transcription-induced supercoils govern their recruitment at genomic sites. Distribution profiles revealed co-localization of RNAP and the two topoisomerases on the active transcriptional units (TUs). At a given locus, topoisomerase I and DNA gyrase were localized behind and ahead of RNAP, respectively, correlating with the twin-supercoiled domains generated. The recruitment of topoisomerases was higher at the genomic loci with higher transcriptional activity and/or at regions under high torsional stress compared to silent genomic loci. Importantly, the occupancy of DNA gyrase, sole type II topoisomerase in *Mtb*, near the Ter domain of the *Mtb* chromosome validates its function as a decatenase.

## Author summary

The generation of DNA topological constraint is intrinsic to transcription. Although *in vitro* studies indicated DNA gyrase and topoisomerase I action in the removal of excess supercoils, ahead and behind the transcribing RNA polymerase, *in vivo* recruitment and interaction of both topoisomerases with the genome has not been investigated. Using advanced sequencing, we have mapped the genome-wide footprints of topoisomerase I and DNA gyrase along with RNAP in deadly pathogen, *Mycobacterium tuberculosis*. We show that *in vivo* distribution of topoisomerases is guided by active transcription and both the enzymes co-occupy active transcription units. We establish their interaction with the regions of genome having propensity to accumulate negative and positive supercoiled domains, validating their role in managing the twin supercoiled domains.

## Introduction

Translocation of the transcription machinery along the duplex DNA molecule causes axial rotation of the DNA duplex relative to the RNAP complex. Consequently, the structural complexity, topography and helical tension on the chromatin result in waves of positive supercoils in front of an advancing polymerase and accumulation of negative supercoils behind. The twin supercoiled domains [1] thus generated and the associated torsional strain are relieved by the action of different classes of topoisomerases [2]. Genetic and *in vitro* studies in *Escherichia coli* have implicated that negative supercoils are acted upon by relaxases i.e. Topo I (Type I) and Topo IV (Type II) while the positive supercoils are removed by the activities of DNA gyrase and Topo IV [2–4]. In parallel, the resolution of sister chromatids is carried out by stronger decatenation activity of Topo IV and assisted by a rather meager activity of DNA gyrase and Topo III [5–7].

Earlier studies aimed to unravel the influence of supercoiling and topoisomerases in the transcription process revealed their close connection [5, 8–10]. A deficiency in the function of topoisomerase activities affects the supercoiling balance [11] resulting in inefficient transcription [12, 13]. The genome-wide gene expression studies have linked the respective relaxation and supercoiling activities of Topo I and DNA gyrase in the modulation of gene expression profile, highlighting the global regulatory roles of topoisomerases [14–16]. However, the mechanism of their *in vivo* influence on gene expression is not understood due to inadequate information about their interaction with the genome. Direct demonstration of their occupancy on different genomic territories is not yet carried out which is a pre-requisite in determining their impact on supercoiling gradients generated in transcriptionally active regions spanning whole genome. Although low resolution genome-wide occupancy of DNA gyrase [17] and more recently that of Topo IV has been revealed in *E*. *coli* [18], genomic landscape of both DNA gyrase and Topo I during transcription has not been elucidated on any bacterial genome so far. In order to achieve such comprehensive view of topoisomerase dynamics, function and factors affecting their distribution, we have deciphered *in vivo* genome-wide binding profile of both classes of topoisomerases together taking the advantage of non-redundancy of topoisomerases in *Mycobacterium tuberculosis* (*Mtb*) genome.

Analysis of the *Mtb* genome [19] indicated the presence of a single Type I (DNA topoisomerase I or Topo I) and a single Type II enzyme (DNA gyrase). Notably, the genome is devoid of any dedicated decatenase gene unlike *E*. *coli* and many other eubacteria. The presence of a single relaxase and a sole supercoiling enzyme in *Mtb* imposes on them the entire burden of managing the topological perturbations occurring during all DNA transaction processes. Accordingly, our earlier studies revealed that mycobacterial gyrase is a strong decatenase [20] and Topo I relaxation activity is stimulated by the nucleoid associated protein HU [21]. The absence of additional topoisomerases with overlapping or back-up function in *Mtb* (unlike in *E*. *coli*) was exploited in the present study to probe the *in vivo* topology-transcription dynamics. We have carried out the first genome-wide Chromatin Immunoprecipitation (ChIP) of both Topo I and DNA gyrase followed by sequencing (Seq) of the bound target DNA. ChIP-Seq analysis revealed the co-localization of Topo I, DNA gyrase and RNAP throughout the *Mtb* genome. We show that the distribution landscape of Topo I and DNA gyrase in the genome is driven by transcription induced supercoiling. We also demonstrate the recruitment of DNA gyrase at Ter region of the chromosome to carry out decatenation function.

## Results

### Genome-wide association of RNAP, Topo I and DNA gyrase

**Fig 1:**
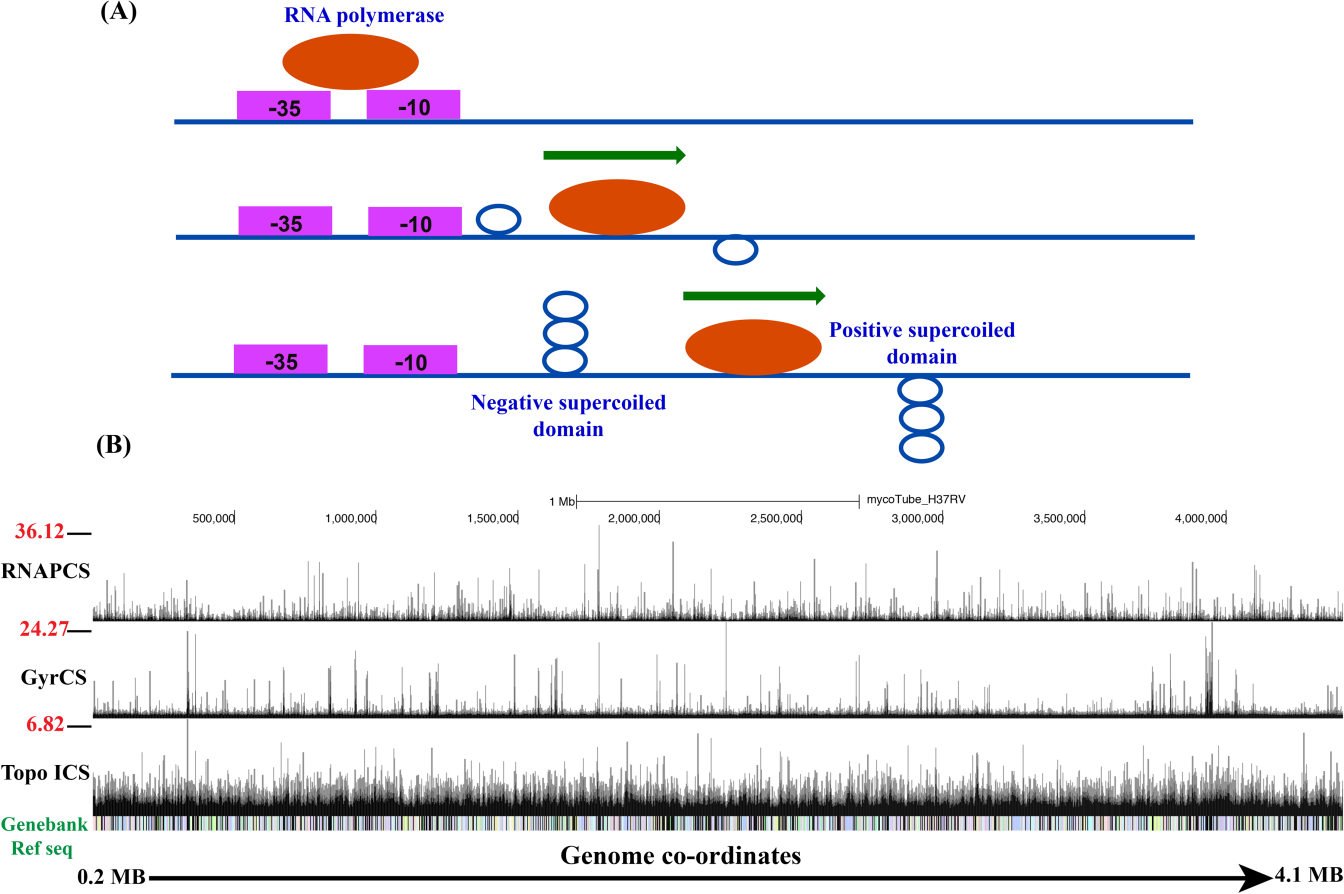
Topoisomerases occupancy on *Mtb* genome. **(A)** Twin supercoiled domain model. The movement of RNA polymerase machine on the genome/TUs generates wave of negative supercoils upstream and positive supercoils downstream to the transcription machine. The supercoils have to be removed by the action of topoisomerases allowing the unobstructed translocation of RNAP. The *in vivo* interaction of topoisomerases with the twin supercoiled domains is yet to be demonstrated. **(B)** ChIP-Seq analysis of RNA polymerase (RNAPCS), DNA gyrase (GyrCS) and Topoisomerase I (Topo ICS) occupancy on *Mtb* genome. UCSC genome browser view of Topo I, RNAP and DNA gyrase occupancy across the *Mtb* genome (0.2 Mb-4.1 Mb representative region of *Mtb* genome). The peak height correspond to the signal intensity (IP/Mock ratio of RNAP, Gyrase and Topo I IPs) of protein binding on the particular site.

Transcription generates waves of positive supercoils downstream and negative supercoils upstream of moving RNAP (Fig 1A). The catalytic activities of Topo I and DNA gyrase ensure the removal of the resultant negative and positive supercoils, respectively. To investigate the interaction of topoisomerases (both Topo I and gyrase) with the *Mtb* genome, the global occupancy of Topo I and DNA gyrase together with RNAP was monitored by ChIP-Seq. Visualization of ChIP-Seq data of RNAP, Topo I and DNA gyrase on UCSC genome browser (Fig 1B and S2 Fig) revealed the distribution of Topo I, Gyrase and RNAP signals throughout the genome. As reported previously, the strongest RNAP signal was present at the putative promoter regions of the TUs [22]. Comparison of Topo I and DNA gyrase signals with the RNAP signals suggested their co-localization throughout the *Mtb* genome.

Based on the peak detection criteria (enrichment ratio; ER>2.0), 190 peaks for Topo I and 73 peaks for DNA gyrase were identified, distributed throughout the genome. The classification of the loci enriched with Topo I and DNA gyrase suggested that the peaks were distributed in various functional groups (S3 Fig). Importantly, the genes occupied by topoisomerases were transcriptionally active [22].

### Identification of Topo I and gyrase DNA binding motifs

The MEME suite [23] was used to identify consensus sequences for Topo I and DNA gyrase from ChIP-Seq signals by scanning 300 bp encompassing the peaks (S4A Fig). The gyrase binding motif was found to be GC-rich compared to the Topo I motif, indicating that the Topo I motif was relatively more prone to DNA melting. FIMO (Find Individual Motif Occurrences) detected 5299 and 9997 motifs (p>0.0001) for Topo I and DNA gyrase respectively. The genome-wide distribution of Topo I and DNA gyrase binding motifs indicates that these enzymes can potentially bind throughout the genome (S4B Fig).

### Topo I and DNA gyrase distribution follow RNAP footmark at TUs

**Fig 2:**
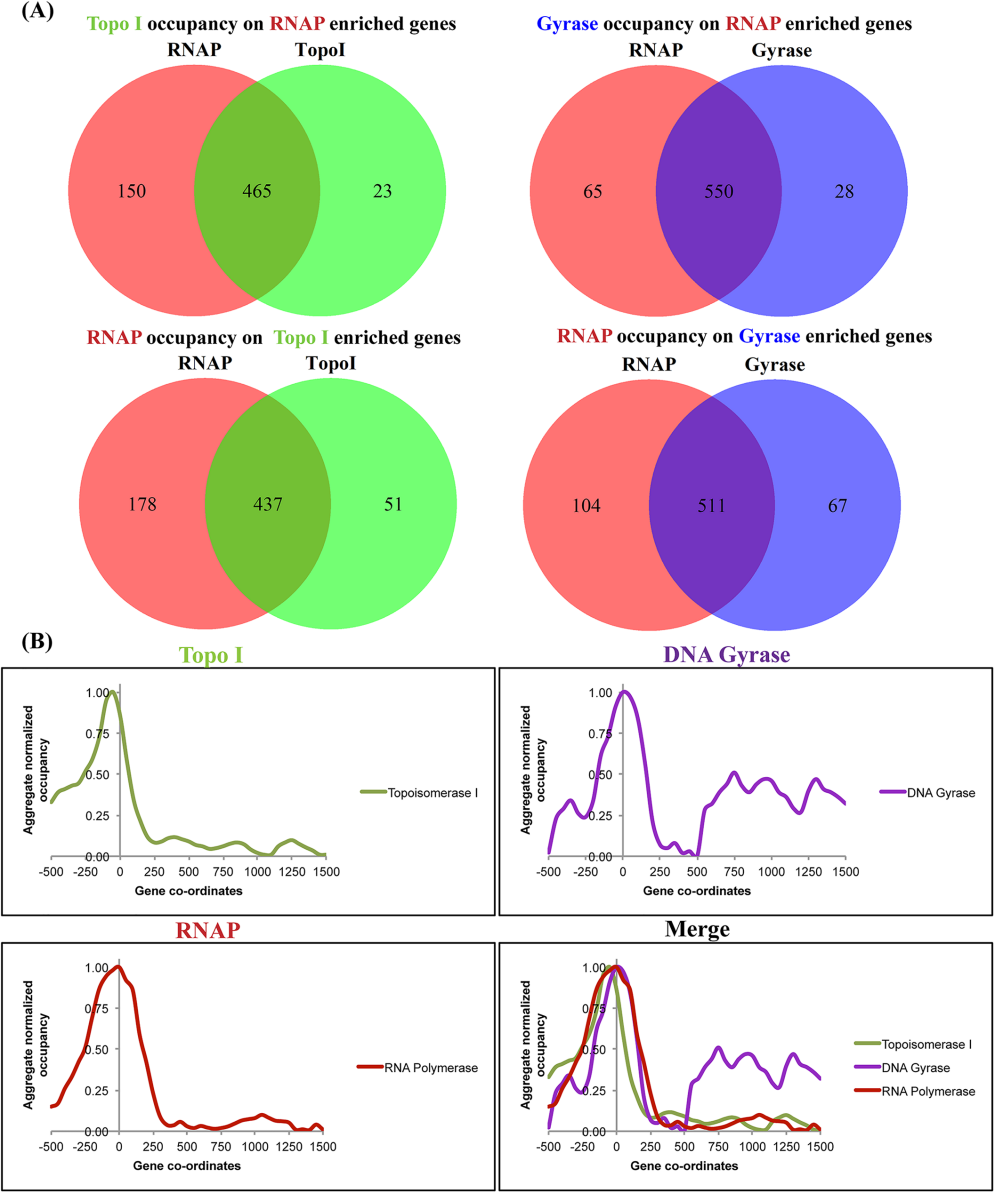
Topo I, RNAP and DNA gyrase co-localize on TUs. **(A)** TopoI, gyrase and RNAP association. Enrichment ratio (ER) for Topo I, Gyrase and RNAP was calculated on protein coding genes as described earlier [22] and in Materials and Methods. RNAP and topoisomerase enriched genes (ER>2) were monitored for the enrichment of Topo I/DNA gyrase and RNAP respectively. The number of genes enriched with both RNAP and topoisomerases are represented at the intesection of venn diagram **(B)** Distribution profile of Topo I, RNAP and DNA gyrase across the TUs. Read counts from transcriptionally active (RPM>1) protein-coding TUs >1 kb in length (N = 541) were taken with reference to the TSS and mean read counts were calculated in 50 bp sized bins. Data were normalized to the input samples followed by removal of background using the read counts associated with the genes with no expression (RPM<1). Data were normalized with the maxima to generate the pattern of distribution on TUs.

The UCSC genome browser view of the binding profile of Topo I, RNAP and DNA gyrase showed that at several places the peaks of these proteins were co-localized suggesting the association of topoisomerases with the RNAP enriched genes (Fig 1B). To analyse further, the enrichment ratio (ER) across the protein coding genes were calculated by taking the ratio of mean read counts of ChIP-samples and Mock IP (control) as described [22]. RNAP enriched (ER>2) genes were monitored for Topo I and gyrase enrichment (ER>2). Data depicted in Fig 2A indicate that out of 615 RNAP enriched genes, 75.6% and 89.0% genes were also enriched for Topo I and DNA gyrase respectively. Moreover, out of 488 Topo I enriched (ER>2) genes, 89.5% were enriched for RNAP while 88.5% gyrase (ER>2) enriched genes were also enriched with RNAP (Fig 2A). The analysis indicated that a majority of the RNAP bound regions exhibit association with the Topo I/DNA Gyrase and *vice versa*.

In order to delineate the global distribution profile of Topo I, RNAP and gyrase across the TUs, all the transcriptionally active TUs (>1 kb length) with identified TSS were selected and the mean read counts around the TSS of all TUs were calculated. The start site (TSS) was taken as a reference point and regions 500 bp upstream and 1500 bp downstream were included to generate the binding profile for Topo I, RNAP and DNA gyrase. The mean of the read counts was plotted to generate a single profile representing the global TU. The strongest peak of RNAP was located around the TSS. The position of the RNAP matches with the previously reported promoter proximal peak [22]. On the same TU, the occupancy signals of Topo I and DNA gyrase were found to be in a close proximity, overlapping to the RNAP peak (Fig 2B). Importantly, around 500 bp downstream of the TSS stronger accumulation of DNA gyrase is apparent which is indicative of its role in removal of excessive positive supercoils generated downstream of the advancing RNAP. Further, analysis of the Topo I and DNA gyrase profile on the TUs depleted of RNAP signals (ER<1) did not exhibit Topo I and gyrase peak around the TSS (S5A Fig) indicating that RNAP activity at TUs facilitates the recruitment of the two topoisomerases.

### Topoisomerases associate with the transcriptionally active TUs

**Fig 3:**
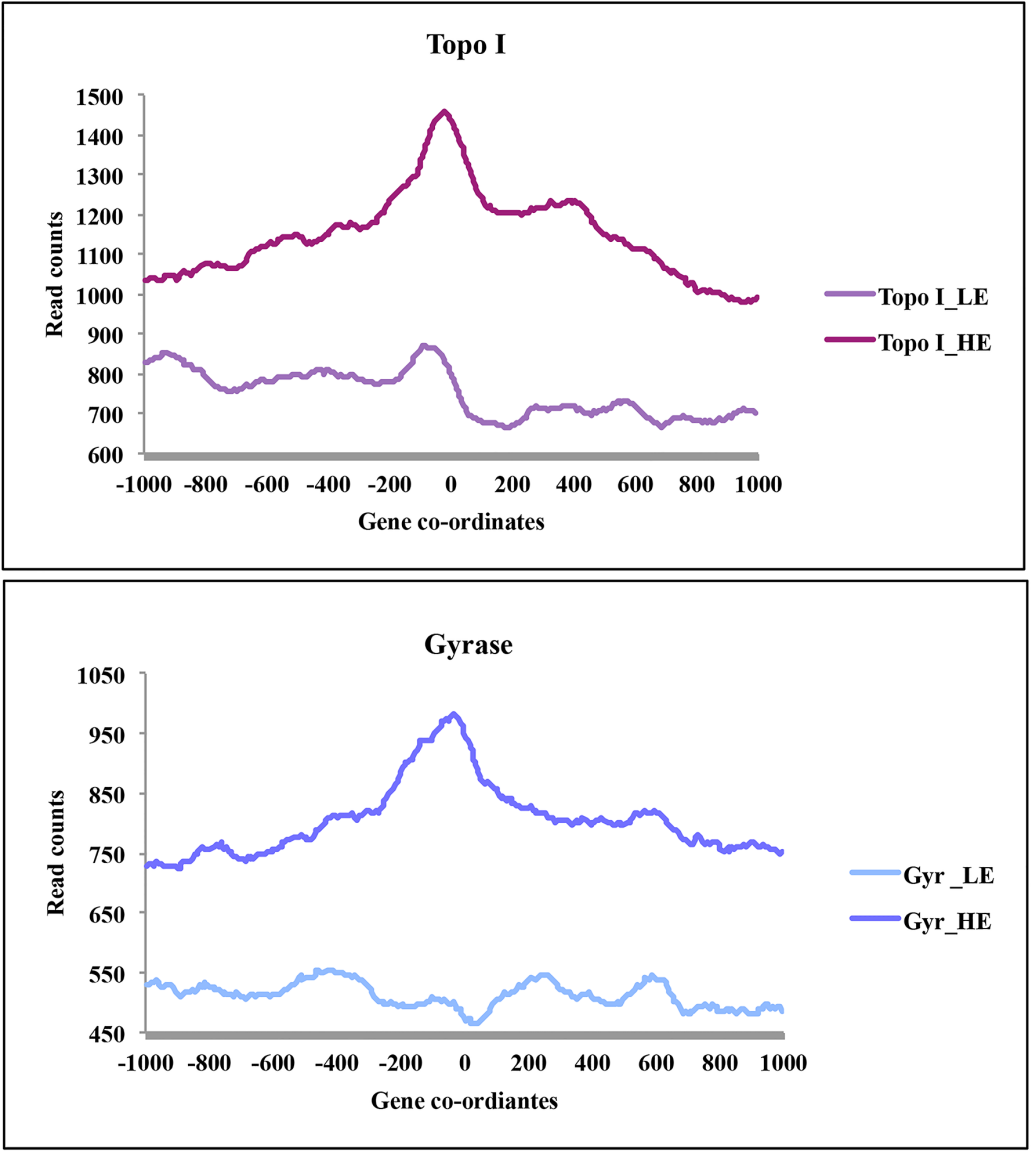
Topoisomerase occupancy varies on genes with different expression levels. The genes were segregated based on the available RNA-Seq data [22] under highly expressed (HE) and lowly expressed (LE) category (N = 342). The HE and LE class of gene co-ordinates were analyzed for the occupancy of topoisomerases. TSS (0) was taken as a reference point and mean read count was plotted around it to generate occupancy profile. Mean read counts corresponding to gene co-ordinates were plotted at a single nucleotide resolution.

**Fig 4:**
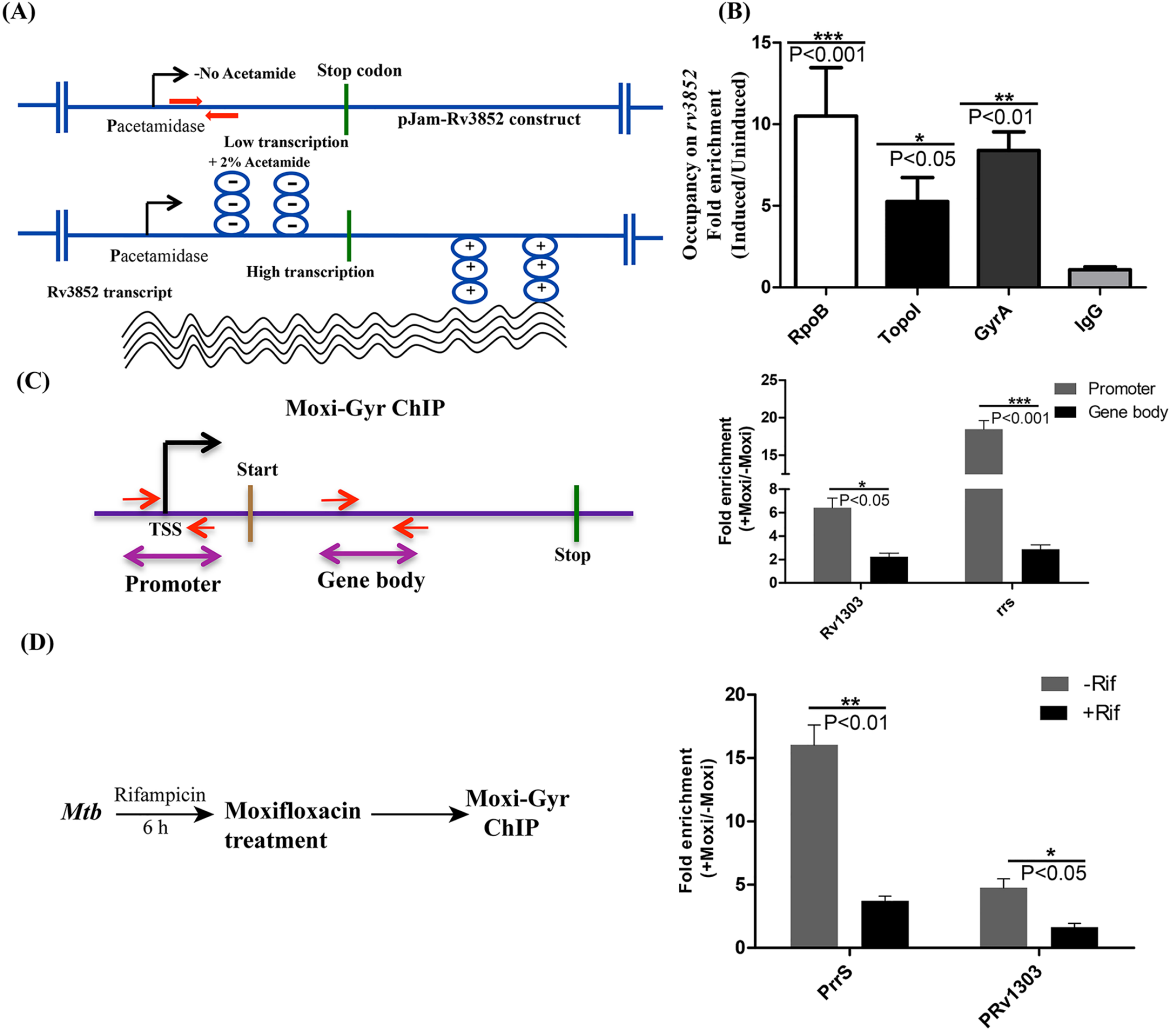
Transcription induction recruits topoisomerases to TU. Determination of the occupancy of RNAP, Topo I and DNA gyrase on activated TU. **(A)** Schematic of the experimental set up. Induction of transcription introduces supercoils on the template which recruits topoisomerases. Construct pJam-Rv3852 was electroporated into *M*. *smegmatis* and the transformants were grown up to the exponential phase. Cultures were induced with acetamide for 6 h to activate the transcription of *rv3852* cloned under the acetamidase promoter. Arrows (red) indicate the position of primers for the amplification of target region **(B)** Induced (I) and uninduced (UI) cultures were processed for ChIP and enrichment of RNAP, Topo I and DNA gyrase was monitored by the qPCR using *rv3852* specific primers. Unrelated IgG antibody was used as a negative control. **(C)** Depiction of the architecture of TU and the positions of the primers used for monitoring the gyrase binding on promoter region (P) and gene body of *Rv1303* and *rrS* TUs. *Mtb* cells were treated with Moxifloxacin (Moxi) and gyrase-DNA clevage complexes around promoter and gene body were detected by qPCR. **(D)** Effect of transcription inhibition on topoisomerase activity. *Mtb* cells were treated with or without Rifampicin (Rif) followed by treatment with Moxifloxacin to induce gyrase-DNA cleavage complex formation. Promoter regions of *Rv1303* and *rrS* were monitored for the formation of gyrase-DNA cleavage complex in the presence and absence of Rif. Error bars represent the SD obtained from three independent experiments. Significance of the observations was assessed by applying unpaired t-test (* = P<0.05; *** = P<0.001; **< P<0.01; ns = not significant).

The transcriptional activity of RNAP would induce torsional stress on the active TUs, which may facilitate the recruitment of topoisomerases. Co-localization of Topo I and gyrase signals genome-wide with RNAP peaks and TUs suggested that topoisomerases were predominantly recruited to active TUs. Investigation of Topo I and DNA gyrase signals on RNAP enriched genes (Fig 2A) further hinted their stronger association with transcriptionally active genes. To get the global view of the relationship between gene transcription and topoisomerase occupancy, TUs were segregated into two categories based on their gene expression profile (based on Reads Per Kilobase of transcript per Million mapped reads i.e. RPKM values), high expression (RPKM>3) and low expression (RPKM<1). A total of 342 protein coding TUs (excluding rRNA and tRNA clusters) from each category were taken and the average occupancy profile of Topo I, DNA gyrase and mock IP was generated (Fig 3 and S5B Fig). From the averaged profiles, it is apparent that both Topo I and DNA gyrase associate strongly with highly expressed genes compared to the genes with low transcriptional activity (Fig 3). Negative control (mock IP) profile did not exhibit any appreciable differences in the enrichment on high and low expression genes (S5B Fig) indicating the specific preferential enrichment of topoisomerases on transcriptionally active genes. To obtain independent confirmation, the *rv3852* gene [24] was cloned under an inducible acetamidase promoter [25] and introduced into *M*. *smegmatis* cells which lack the ortholog. The advantages of using this experimental set up were (a) the transcription of the TU can be induced strongly by the addition of acetamide, (b) the higher level of transcription achieved in this system would lead to accumulation of supercoils, (c) the absence of the orthologous gene allows monitoring of the occupancy of the topoisomerases and RNAP on the transcriptionally active supercoiled template without any interference from the genomic copy. Exponential phase cells were induced to activate transcription of *rv3852* (Fig 4A) and the occupancy of RNAP, Topo I and DNA gyrase was monitored by ChIP-qPCR. From Fig 4B, it is evident that upon activation of transcription, topoisomerases were recruited to the *rv3852* TU. To confirm the recruitment of topoisomerases to transcriptionally active DNA template, we have selected two TUs *rrS* and *Rv1303* TUs, which were found to be transcriptionally active. On these TUs functional gyrase association was monitored by trapping the active gyrase molecules with moxifloxacin followed by monitoring its enrichment around promoter region as well on gene body. At both the regions moxifloxacin trapped gyrase-DNA cleavage complexes were observed (Fig 4C). To establish that the functional gyrase-DNA complex formation is governed by transcription, *Mtb* cells were treated with sub-lethal concentrations of Rifampicin to inhibit transcription. Inhibition of transcription led to the reduction in ChIP-enrichment of DNA gyrase validating the model that transcription governs the recruitment of topoisomerases (Fig 4D). *In vitro*, validation of the hypothesis was carried out by monitoring the recruitment of topoisomerases on the transcriptionally active DNA template. Recruitment of both DNA gyrase and Topo I on plasmid DNA was significantly higher when transcription was activated by the addition of RNAP and NTPs compared to the reaction where transcription did not occur (S6 Fig). Overall, ChIP-Seq analysis, *in vitro* transcription and *in vivo* gene expression studies confirm the recruitment of topoisomerases to transcriptionally active DNA template.

### Topoisomerases are recruited to supercoiled domains

**Fig 5:**
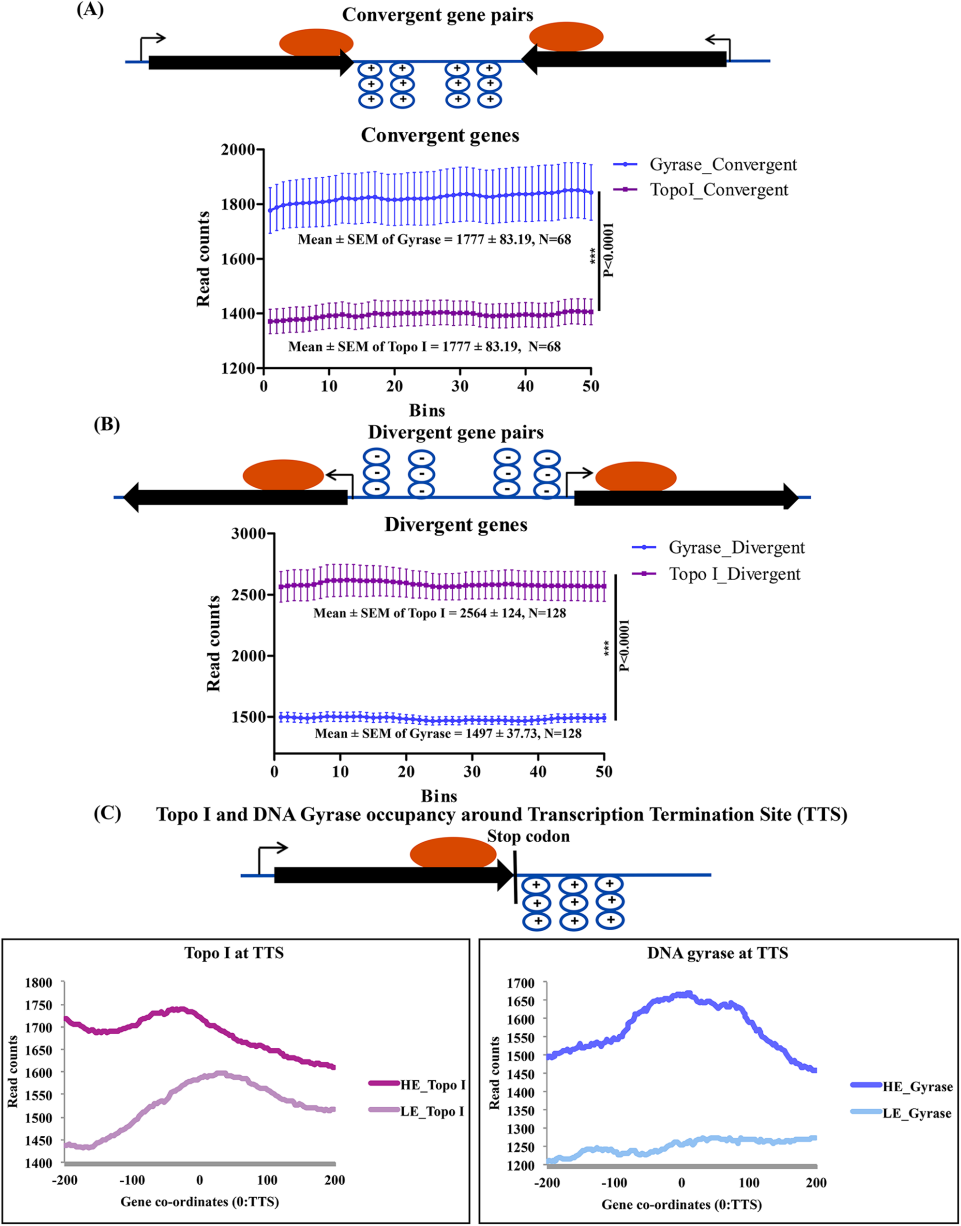
Transcription induced supercoils recruit topoisomerases. **(A)** and **(B)** Occupancy of topoisomerases on convergent and divergent genes respectively. The convergent (N = 68) and divergent (N = 128) gene pairs were extracted (from NCBI) and the intergenic region between the gene pairs was divided into 50 equal sized bins and the number of reads in every bin were averaged and mean values were plotted to monitor the occupancy of Topo I and DNA gyrase. Significance of the observations was tested by applying unpaired t-test (*** = P<0.0001) **(C)** The occupancy of topoisomerases downstream to the transcription termination sites (TTS) of HE and LE genes (N = 342). Based on the RNA-Seq profile, genes with Low (LE) and High expression (HE) were segregated and their predicted TTS was used as a reference point for the analysis. The mean read counts were calculated around the TTS (-200 bp to +200 bp) and plotted at a single nucleotide resoltion to generate the occupancy profile of DNA gyrase and Topo I.

To study the interaction of topoisomerases with supercoiled domains, the occupancy of topoisomerases was determined on the intergenic regions of the convergent and divergent gene pairs. In the convergent gene pairs, the head-on movement of RNAP would induce positive supercoils downstream of both genes while movement of RNAP in the opposite direction would result in the accumulation of negative supercoils upstream of divergent gene pairs. Thus, the intergenic regions between convergent and divergent gene pairs would harbor distinct supercoiled domains generating the sites for the recruitment of respective topoisomerases. Convergent and divergent gene pairs were segregated and the occupancy of Topo I and DNA gyrase was monitored in the intergenic regions (Fig 5A and 5B). In every gene pair at least one of the TU was transcriptionally active (RPKM>1). The analysis revealed higher occupancy of DNA gyrase in the intergenic regions between convergent genes (Fig 5A) whereas a higher density of Topo I was seen in the intergenic regions between the divergent genes (Fig 5B). The twin-supercoiled domain model also predicts the accumulation of positive supercoils downstream of the region where transcription termination occurs and RNAP dissociates [26] implying that DNA gyrase should be recruited to the end of TUs. In order to test this hypothesis, employing RNA-Seq data [22] we have identified the putative transcription termination site (TTS) of the highly expressed and lowly expressed TUs. Around the TTS, 200 bp upstream and downstream regions were scanned for the occupancy of Topo I or DNA gyrase. From the data presented in Fig 5C, it is apparent that the occupancy of DNA gyrase is high downstream to TTS. Notably, the higher enrichment of DNA gyrase on the highly expressed genes compared to the lowly expressed genes suggested that the enrichment resulted from the accumulation of torsional stress generated due to active transcription. In contrast, Topo I enrichment peak was seen upstream of the DNA gyrase peak around TTS. The pattern of Topo I and DNA gyrase occupancy at the end of TUs establishes the existence of twin-supercoiled domain *in vivo*. Thus, overall, it is evident that Topo I and DNA gyrase are distributed on active TUs being recruited to negatively and positively supercoiled domains, respectively.

### Global landscape of Topo I and DNA gyrase on *Mtb* chromosome

**Fig 6:**
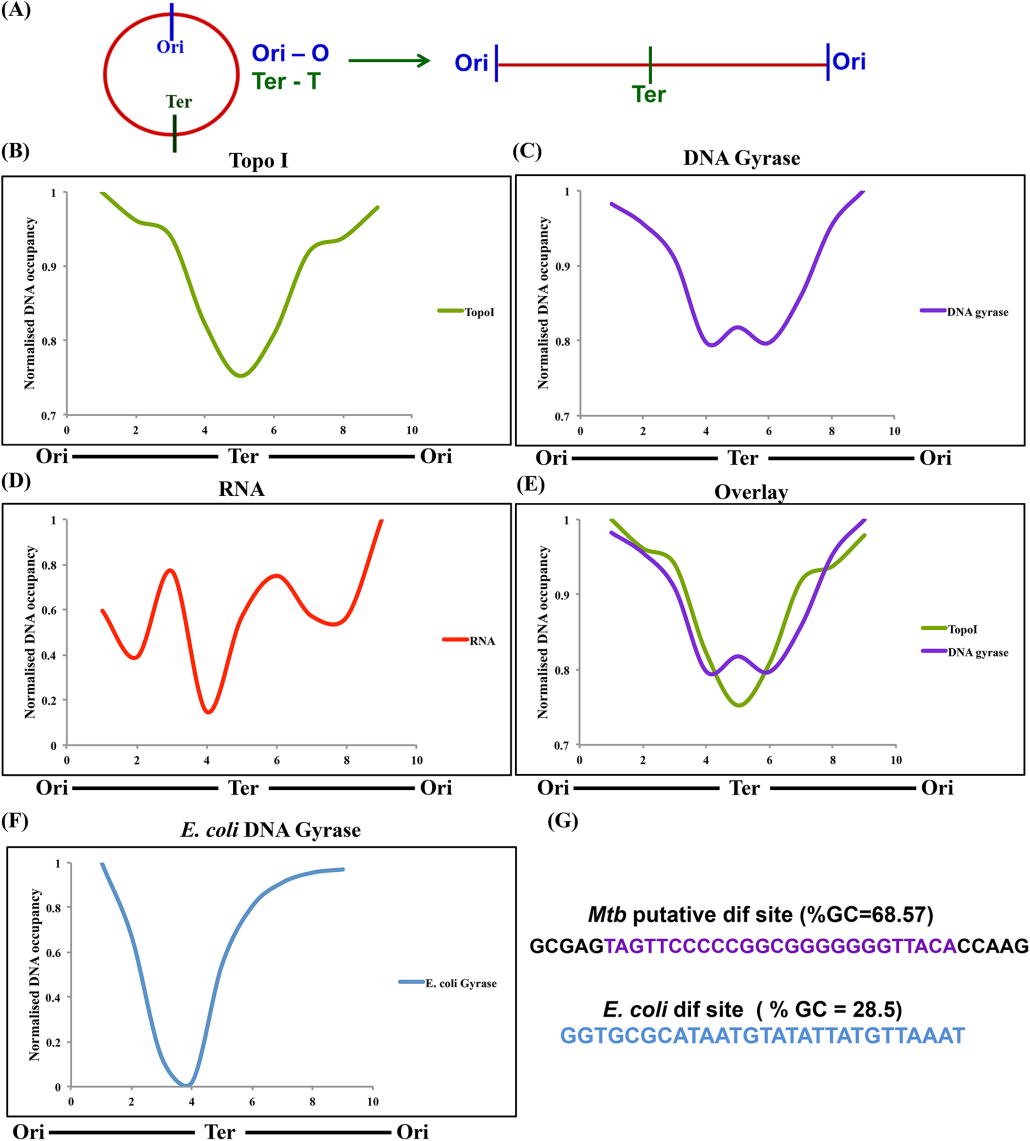
Genome-wide distribution profile of Topo I, DNA gyrase, RNA polymerase and transcript abundance. *Mtb* genome was divided into 9 bins (~ 500 kb) and mean read counts in each bin were calculated. The data were normalized to the input and plotted. The plots represent the linear genome expanding from left *ori* (O) to the right *ori*. **(A)** Relative localization of *ori* and *ter* (T) region on circular and linear genome. Occupancy profile of **(B)** Topo I **(C)** DNA gyrase **(D)** Transcript abundance across the genome of *Mtb* [22] **(E)** Overlay of Topo I and DNA gyrase **(F)** Occupancy profile of *E*. *coli* DNA gyrase derived from the ChIP-ChIP data [17] **(G)** Depiction of putative *Mtb dif* site sequence derived from the gyrase peak at Ter domain.

In terms of transcriptional activity, the bacterial genome is non-uniform since some regions of the genome have higher transcriptional activity compared to others [22]. This may influence genome-wide supercoiling and thus the distribution of DNA topoisomerases across the genome. In order to explore their genomic distribution, the *Mtb* genome was divided into 9 bins (~ 500 Kb) and the associated read counts were averaged for each bin (Fig 6A). This revealed the non-random occupancy profile of topoisomerases across the genome. The association of DNA gyrase and Topo I was found to be higher at the OriC region of the genome compared to the other parts indicating enhanced torsional stress there (Fig 6B and 6C). To establish whether higher torsional stress at OriC and thus higher occupancy of the topoisomerases is an attribute of transcription, transcript abundance data were plotted (Fig 6D). The pattern of transcript abundance was found to correlate with the occupancy profile of the topoisomerases thus reconfirming that higher transcriptional activity across the genome recruits topoisomerases.

Comparison of the occupancy profile of *Mtb* DNA gyrase with that of Topo I indicated a stronger occupancy of DNA gyrase near the Ter region of the genome although at OriC both the Topo I and DNA gyrase were abundant (Fig 6E). At the end of DNA replication, daughter DNA molecules are entangled at the Ter domain. These entangled DNA molecules are resolved by the decatenation activity of Type II topoisomerases [27, 28]. Mycobacterial DNA gyrase has evolved a strong decatenation ability [20, 29, 30] in addition to its characteristic supercoiling function. In contrast to *Mtb*, *E*. *coli* DNA gyrase has poor decatenation activity and the decatenation function is carried out by Topo IV [4]. Studies with *E*. *coli* [18, 31] have shown the norfloxacin-mediated entrapment of catalytically active Topo IV at the genomic *dif* site which is required for the de-catenation function *in vivo* [18]. The comparative analysis of the binding profile of *Mtb* and *E*. *coli* DNA gyrase [17] showed the recruitment of gyrase from both species to the respective OriC regions (Fig 6E and 6F). However, only *Mtb* gyrase was specifically recruited at the Ter region (Fig 6E). Sequence analysis of the *Mtb* gyrase peak at Ter domain indicated the presence of GC rich sequence (Fig 6G). Comparison of this putative *dif* site with the *E*. *coli dif* sequence and other *dif* sequences [32] did not indicate any conservation with the mycobacterial sequence (Fig 6G). From these data it is apparent that the binding of *Mtb* DNA gyrase at the Ter region could be associated with its decatenation activity *in vivo*.

## Discussion

**Fig 7:**
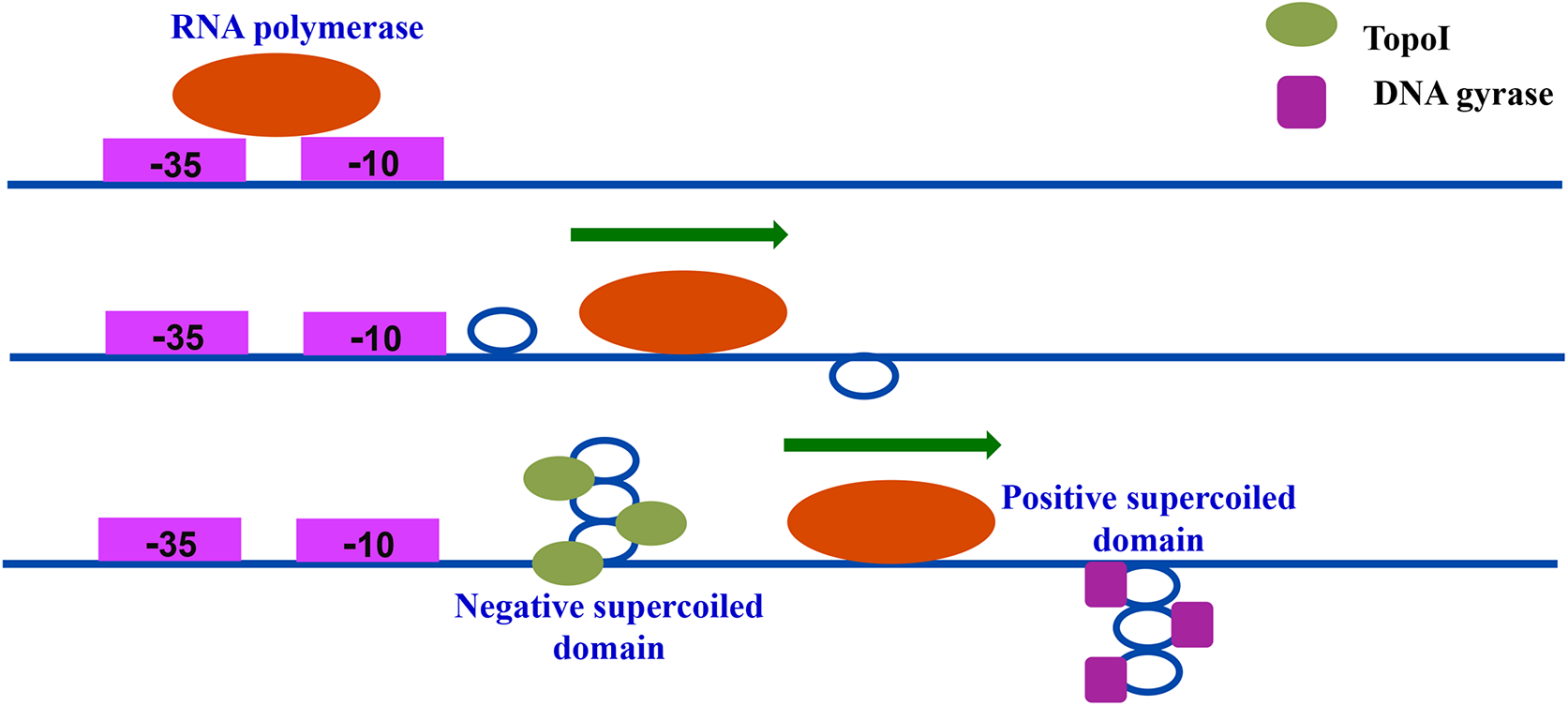
DNA topoisomerases interact with the supercoiled domain on transcriptionally active TUs. Advancement of RNA polymerase on DNA template generates waves of positive supercoils ahead and negative supercoils behind (Twin-supercoiled domain). Topo I is recruited to sites where negative supercoils are prominent while DNA gyrase occupies the region with positive supercoils. Together these enzymes maintain the transcription template topology for optimal transcription.

In 1987, Liu and Wang proposed that the active transcription machine generates two supercoiled domains in the DNA template during transcription [1]. The proposal was based on the rationale that movement of the bulky transcriptional complex of RNAP along a DNA duplex would lead to the rotation of the DNA rather than of RNAP. This model predicted the generation of waves of positive supercoils downstream and accumulation of negative supercoils upstream of the advancing transcription apparatus. Several *in vitro* studies showed that transcription alters DNA template topology thus supporting the twin-supercoiled domain model [33–35]. Next, various DNA sensors of supercoiling were used to assess the operation of twin-supercoiled domain model *in vivo* [26, 36]. The B-to-Z structural transitions of (CG) tracts, and the Tn3 and γδ resolution assays were used as supercoiling probes to examine transcription induced local topological changes [26, 36]. That the transcription induced supercoils are acted upon by Topo I and DNA gyrase to permit the continuation of the process was indicated by the inhibition of DNA gyrase, which led to the accumulation of positively supercoiled DNA [37], and by mutation of Topo I, which generated negatively supercoiled DNA [38]. Based on these studies, it was proposed that Topo I acts upstream while DNA gyrase functions downstream of the RNAP machinery. However, direct evidence for the interaction of topoisomerases with the twin-supercoiled domains was not available until the present study. The genome-wide occupancy profile of topoisomerases along with RNAP in *Mtb* described here is the first demonstration of the interaction of topoisomerases with the twin-supercoiled domains *in vivo* (Fig 7). Our genome-wide distribution of Topo I and DNA gyrase data and their binding profiles correlate well with that of RNAP indicating that there is a functional association of RNAP and topoisomerases. The interaction of topoisomerases with the transcription machinery may recruit them to the site of transcription where topoisomerase activity is needed to foster transcription. In *E*. *coli*, physical association of RNAP with Topo I was revealed [39]. Similarly, in mycobacteria, DNA gyrase was found in a complex with RNAP [40]. Thus, the co-localization of genome-wide peaks of two topoisomerases that carry out two opposing topological reactions, along with RNAP, implicates their *in vivo* role in the stimulation of transcription.

Returning to the twin-supercoiled domain model, it is imperative that the transcriptional activity of RNAP generates supercoils in the vicinity of RNAP, which then has to be removed by the action of topoisomerases suggesting that these three players should work together. Indeed, the co-occupancy of topoisomerases and RNAP on the TUs indicated the co-existence of these proteins. Promoter proximal peaks of RNAP reflect the transcriptional complex, which assembles and pauses at the beginning of genes [22, 41–43]. Once RNAP has entered the elongation phase, it will have generated negative supercoils upstream and positive supercoils downstream. From the binding profiles, it is apparent that the Topo I occupancy is higher upstream of RNAP while the gyrase occupancy peak was found to be present either along with RNAP or downstream of it within the ORF. Thus, the interaction of topoisomerases with the twin-supercoiled domains on a genome-wide scale is evident. Topoisomerase recruitment was found to be enhanced by transcription induction while the inhibition of transcription resulted in decreased gyrase (present study) and Topo IV activity [18] suggesting that the rate of transcription is the major factor governing the chromosomal supercoiling levels. An earlier study in *E*. *coli* and *Salmonella* [11] also indicated the role of RNAP elongation rate in generation of supercoils on the highly expressed genes. Overall, the enhanced association of topoisomerases with the TUs driving active transcription (which would generate high torsional stress) further validates the twin-supercoiled domain model.

Transcriptome analysis of various yeast topoisomerase mutants demonstrated that the transcript abundance of genes with higher transcriptional activity was specifically reduced suggesting that activities of topoisomerases facilitate the transcription of genes [44]. Since DNA topoisomerases maintain the topological homeostasis inside the cell, perturbation of their activity would lead to changes in genome supercoiling and gene expression thereby highlighting their global regulatory role [14–16]. To carry out the global regulatory functions, topoisomerases interact with DNA and remove the excessive supercoiling thus ensuring balanced supercoiling levels in the cell. The genome-wide distribution of the recognition motifs of topoisomerases implies their interaction throughout the genome. However, the binding profile of Topo I and DNA gyrase indicated their non-random distribution, which is likely a consequence of differential accumulation of torsional stress across the genome. In addition to transcription and replication, various processes involving DNA, such as recombination or chromosome segregation, contribute to the differential distribution of torsional stress. One such epicenter of high torsional stress is the OriC region. The distribution profile of topoisomerases indicates their higher occupancy at OriC compared to the Ter domain. At OriC region, the replication machinery binds, melts the DNA and then initiates replication, which further enhances the torsional stress [45]. In every organism studied so far, higher transcriptional activity occurs at the OriC region [46]. Thus, a combination of the initiation of DNA replication and the higher transcriptional activity at OriC subjects this region to a greater torsional stress requiring the higher activity of topoisomerases.

A contrasting landscape emerges at the Ter region when the distribution of topoisomerase there is compared to that of the TUs and OriC. Instead of a near symmetric distribution i.e. Topo I and gyrase occupancy on either side of the replication or transcription complex, only a gyrase peak is found. At the completion of DNA replication, the entangled daughter DNA molecules have to be segregated at or near the Ter domain of the genome [47, 48]. In *E*. *coli*, resolution of daughter catenated molecules is carried out by the action of Topo IV, a dedicated decatenase while *E*. *coli* DNA gyrase carries out primarily supercoiling function [7, 49]. The enhanced binding of *Mtb* DNA gyrase at the Ter domain seen in the present analysis appears to be a characteristic of *Mtb*; *E*. *coli* DNA gyrase does not show binding to the Ter domain. Our earlier studies revealed that mycobacterial DNA gyrase is a dual function enzyme with efficient decatenation activity apart from its natural supercoiling function [20]. Thus, these data provide the first *in vivo* evidence for the action of DNA gyrase at the Ter region most likely to carry out its efficient decatenation function required for daughter chromosome segregation.

In conclusion, genome-wide ChIP-seq analysis of topoisomerases and RNAP demonstrates the interaction of Topo I and DNA gyrase with the twin-supercoiled domain *in vivo* (Fig 7). The global binding profiles of these players demonstrate their co-existence on the TUs with a non-random, organized distribution, correlating with the organization of TUs along the genome and extent of transcriptional activity. Additional experiments to probe the supercoiling of the genome [50] and topoisomerase occupancy by genetic or chemical perturbation of topology would provide deeper insights into the dynamics of supercoiling and the action of DNA topoisomerases in conjunction with the molecular machines engaged in replication and transcription.

## Materials and methods

### Bacterial strains and culture conditions

*Mtb* H37Rv or *Mtb* H37Ra were grown in Dubos Broth (Difco) supplemented with Middlebrook albumin dextrose catalase (ADC) enrichment and 0.05% Tween 80 or on solid Middlebrook 7H11 medium (Difco) supplemented with oleic acid-albumin-dextrose-catalase (OADC).

### Chromatin immunoprecipitation (IP) experiments

*Mtb* cultures (50 ml) grown to OD_600 nm_ = 0.4–0.6 were treated with formaldehyde (final concentration 1%) and incubated for 10 min at 37°C. Cross-linking was quenched by addition of glycine (final concentration 125 mM). Cells were then collected by centrifugation, washed twice with Tris-buffered saline (20 mM Tris-HCl pH 7.5, 150 mM NaCl) and stored at -80°C. Pellets were re-suspended in 4 ml IP buffer (50 mM HEPES-KOH pH 7.5, 150 mM NaCl, 1 mM EDTA, 1% Triton X-100, 0.1% sodium deoxycholate, 0.1% SDS, Roche Antiprotease mini) and sonicated in a water bath sonicator (Bioruptor, Diagenode) to shear DNA to an average size of 150–700 bp. Cell debris was removed by centrifugation and the supernatant was used as input sample in subsequent IP experiments. The samples were incubated overnight at 4°C on a rotating wheel with antibodies (polyclonal anti-GyrA or monoclonal anti-Topo I [51]. An IP experiment without antibody served as negative control (Mock IP). Protein–DNA complexes were immuno-precipitated with 50 µl of Dynabeads Sheep IgG (Dynal Biotech) for 2 h at 4°C. The magnetic beads were collected and washed twice with IP buffer, once with IP buffer containing 500 mM NaCl, once with wash buffer III (10 mM Tris-HCl pH 8, 250 mM LiCl, 1 mM EDTA, 0.5% Nonidet-P40, 0.5% sodium deoxycholate), and once with Tris-EDTA buffer (pH 7.5). IP complexes were eluted from the beads by treatment with 100 µl elution buffer (50 mM Tris-HCl pH 7.5, 10 mM EDTA, 1% SDS) at 65°C for 20 min. Samples were then treated with 2 µl RNase A (10 mg/ml) and cross-links were reversed by incubation for 2 h at 56°C and 6 h at 65°C in 0.5x elution buffer containing 2.5 µl proteinase K (20 mg/ml). DNA was extracted twice with phenol chloroform, precipitated and re-suspended in 20 µl of water. ChIP-qPCR was carried out to determine the enrichment of target sites for Topo I and Gyrase using specific primers for the expected targets (S1 Fig). After confirming target enrichment, samples were processed for sequencing.

### ChIP-Seq library construction and sequencing

DNA fragments (150 to 250 bp) were selected for library construction and sequencing libraries were prepared using the ChIP-Seq Sample Preparation Kit (Illumina; San Diego, California, USA; Cat. No. IP-102-1001) according to the protocol supplied with the reagents. Prior and post library construction, ChIP products were quantified using a Qubit fluorometer (Invitrogen; Carlsbad, California, USA). One lane of each library was sequenced on the Illumina Genome Analyzer IIx using the Single-Read Cluster Generation Kit v4 and 36 Cycle Sequencing Kit v4. Data were processed using the Illumina Pipeline Software v1.60.

### ChIP-Seq data analysis

ChIP-Seq analysis was performed using the HTS station pipeline (http://htsstation.epfl.ch/). Single-end sequence reads generated from ChIP-Seq experiments were aligned to the *Mtb* H37Rv genome (NCBI accession NC_000962.2) using Bowtie [52] with default option in HTS station [53]. Different BAM files were normalized for sequencing depth. At each base position, number of reads mapping to that base was calculated and normalized to the total number of mapped reads [22]. Peaks were analyzed using MACS v.1.4 [54] with parameters ‘‘-bw 200 -m 10100”. Alignment files were converted to bigWig format for visualization in the UCSC genome browser *Mtb* H37Rv 06/20/1998 assembly [55]. To determine the level of ChIP-Seq enrichment for genes, the enrichment ratio (ER) was calculated by dividing the read counts for the ChIP-Seq sample by the read counts of the Mock IP sample. Topo I and Gyrase binding site motifs were searched for using the MEME Suite (http://meme.nbcr.net/meme/) in regions 300 bp upstream and 300 bp downstream of the predicted peak summit and a consensus motif sequence was deduced. ChIP-Seq data for RNAP was taken from NCBI [22] and analyzed as described above. For generating the occupancy profile, transcription start site (TTS) co-ordinates were obtained from Cortes et al., 2013 [56].

### Genome annotation

*Mtb* H37Rv genome annotation was taken from the TubercuList database (http://tuberculist.epfl.ch/), which contains 4019 protein coding sequence (CDS), 73 genes encoding for stable RNAs, small RNAs and tRNAs.

### *In vivo* recruitment of Topo I and DNA gyrase on activated TU

*Mycobacterium smegmatis* mc^2^ 155 cells were electroporated with the pJAM2-Rv3852 construct [24]. The exponentially grown culture was treated with 2% acetamide for 6 h to induce transcription of the *rv3852* gene cloned under the acetamidase promoter. Treated and untreated cultures were processed for ChIP using RpoB, Topo I and GyrA-specific (against GyrA subunit of DNA gyrase) antibodies. From the purified ChIP DNA, qPCR analysis was carried out to monitor the occupancy of Topo I, DNA gyrase and RNAP on *rv3852*. The data were normalized with the mock control (no antibody) and presented as enrichment over the untreated samples (induced/uninduced). A non-specific (unrelated) IgG antibody was used as a negative control.

For Moxifloxacin (Moxi)-gyrase ChIP (Moxi-Gyrase ChIP), *Mtb* cells were treated with 1.25 μg/ml for 8 h, collected by centrifugation, washed (as described above) and re-suspended in a buffer containing 50 mM HEPES-KOH pH 7.5, 5000 mM NaCl, 1 mM EDTA, 1% Triton X-100, 0.1% sodium deoxycholate, 0.1% SDS, Roche Antiprotease mini) and further processed as described above. The transcription inhibition was carried out by treating *Mtb* cells with 0.25 μg/ml Rifampicin (Rif) for 6 h prior to the treatment with Moxi.

### *In vitro* occupancy of topoisomerases on transcription template

To assess the occupancy of the topoisomerases on DNA engaged in active transcription, *in vitro* transcription reactions were carried out. The reactions were carried out in transcription buffer (50 mM Tris HCl, (pH 8.0 at 25°C), 10 mM magnesium acetate, 100 μM EDTA, 100 μM DTT, 50 mM KCl, 50 μg/ml BSA, 5% glycerol) using 30 nM supercoiled plasmid (*Mtb*Gyr1542-pARN104-harboring the 867 bp region including the gyrase promoter elements and 675 bp region upstream and downstream to the transcription start site of *gyrB* respectively) supplemented with 100 μM of NTPs and with or without 200 nM of σ^A^-enriched RNAP (*M*. *smegmatis*) [57]. Reaction mixtures were incubated at 37°C for 5 min and supplemented with 30 nM of *M*. *smegmatis* Topo I, and 60 nM of reconstituted gyrase from *Mtb*, then incubated further at 37°C for 5 min and cross-linked using a UV trans-illuminator (365 nm for 30 min) followed by ethanol precipitation. The pellet was dissolved in 40 µl of distilled water and treated with DNase I (New England Biolabs, UK). Bound gyrase and Topo I were transferred to nitrocellulose membrane by slot blotting and analyzed by their respective anti-GyrA and anti-Topo I antibodies.

## Supporting information

S1 Fig Co-immunoprecipitation of target bound DNA and detection by qPCR. (A) Fragmentation of *Mtb* DNA used for ChIP. Detection of target DNA sequence of corresponding proteins by qPCR. (B) RpoB subunit of RNAP (C) Topo I (D) Gyrase. P*rpoB* and P*sigA*: Promoter of *rpoB* and *sigA* respectively. *rpoB* ORF: Open Reading Frame or coding region of *rpoB*.
(PDF)

S2 Fig Comparison of ChIP-Seq signals (IP/mock normalised) between *M*. *tuberculosis* Ra and Rv topoisomerases. The pearson co-relation coefficient of ChIP-seq signals between *M*. *tuberculosis* Ra (MtbRaCS) and *M*. *tuberculosis* Rv (MtbRvCS) Topo I was 0.67 while for DNA gyrase was found to be 0.78.
(PDF)

S3 Fig Distribution of Topo I and gyrase peaks across different functional classes of genes. (A) Distribution of Topo I peaks (B) Distribution of gyrase peaks.
(PDF)

S4 Fig Consensus recognition sequence of Topo I and DNA gyrase. (A) Consensus motifs were obtained from the respective peaks using MEME (B) FIMO detected the genome wide distribution of Topo I and DNA gyrase peaks. The genomic co-ordinates of motifs were taken from FIMO and CG view was used to represent the distribution of consensus motifs on circular genome of *Mtb*.
(PDF)

S5 Fig (A) **Topo I and gyrase occupancy profile on genes depleted of RNAP**. TUs with low transcriptional activity (RPM<1) were selected and analyzed for RNAP enrichment and only TUs with RNAP Enrichment ratio (ER<1) (N = 206) were selected for generating the profile of Topo I and DNA gyrase occupancy around the TSS (0). Mean read counts were calculated in 50 bp sized bins. Data were normalized with the maxima to generate the pattern of distribution on TUs. (B) **Evaluation of non-specific ChIP enrichment on highly expressed (HE) genes.** The genes were segregated based on RPM values into highly expressed (HE) and low expressed (LE) category as described in Fig 3. The mean read counts were plotted at a single nucleotide resolution around the TSS (-250 to +250) to generate the occupancy profile.
(PDF)

S6 Fig *In vitro* occupancy of topoisomerases on active transcription template. Supercoiled transcription template harboring *gyrB* promoter and 675 bp transcript coding region were incubated with reaction mix with or without RNAP as depicted in schematic (A). Following the transcription, the accumulated Topo I and DNA gyrase on topologically stressed template were precipitated and detected by immuno-slot blotting (B) and quantification was carried out based on three independent experiments (C). Error bars represent the standard deviation obtained from three independent experiments.
(PDF)
