# Supplementary figures and images for: Transcription facilitated genome-wide recruitment of topoisomerase I and DNA gyrase
Source: PLoS Genet. 2017 May 2;13(5):e1006754. doi: 10.1371/journal.pgen.1006754 (PMC5433769; doi:10.1371/journal.pgen.1006754)

# S1 Fig.

(A)

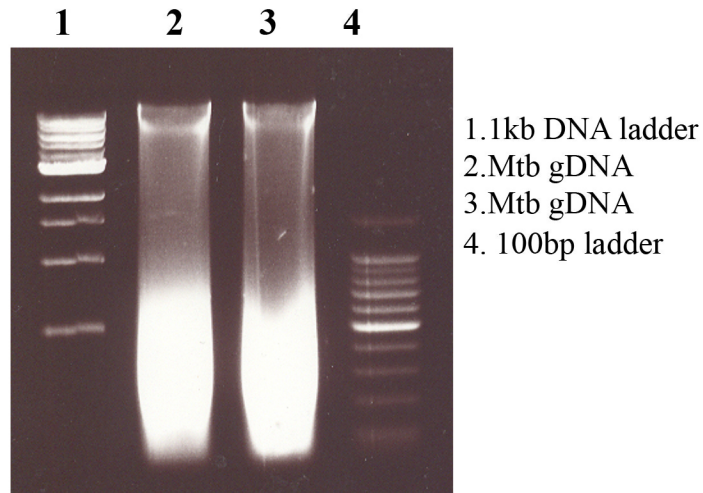

(B)

RpoB

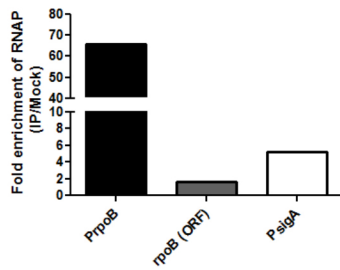

(C)

Topo I

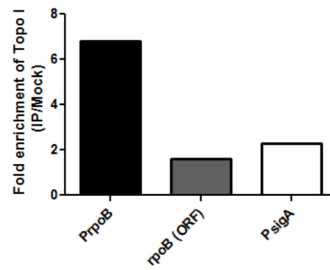

(D)

Gyrase

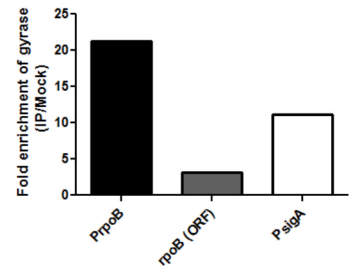

Supplement: S1 Fig — (A) Fragmentation of Mtb DNA used for ChIP. Detection of target DNA sequence of corresponding proteins by qPCR. (B) RpoB subunit of RNAP (C) Topo I (D) Gyrase. PrpoB and PsigA: Promoter of rpoB and sigA respectively. rpoB ORF: Open Reading Frame or coding region of rpoB. (PDF) [file pgen.1006754.s001.pdf]

S2 Fig.

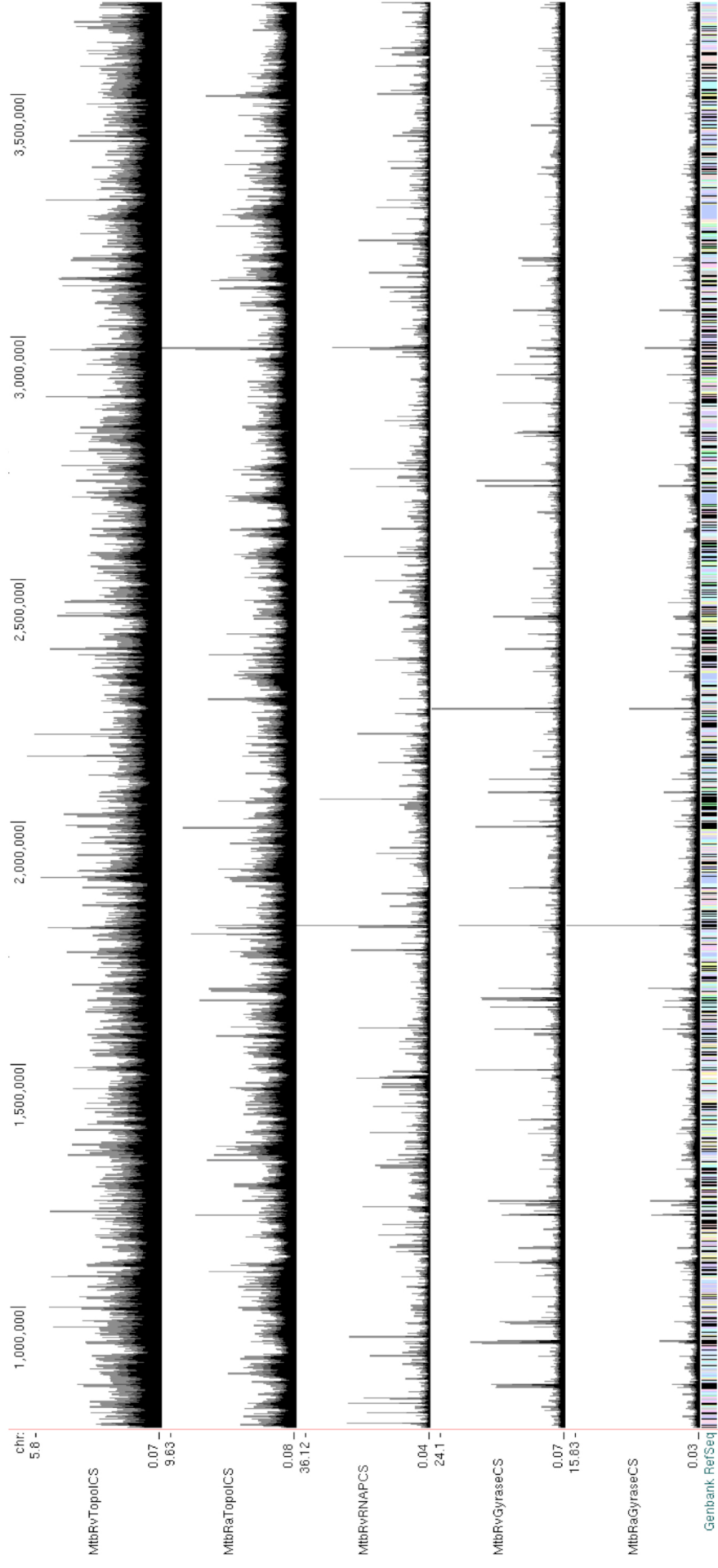

Supplement: S2 Fig — The pearson co-relation coefficient of ChIP-seq signals between M. tuberculosis Ra (MtbRaCS) and M. tuberculosis Rv (MtbRvCS) Topo I was 0.67 while for DNA gyrase was found to be 0.78. (PDF) [file pgen.1006754.s002.pdf]

# S3 Fig.

(A)

Topo I peak distribution

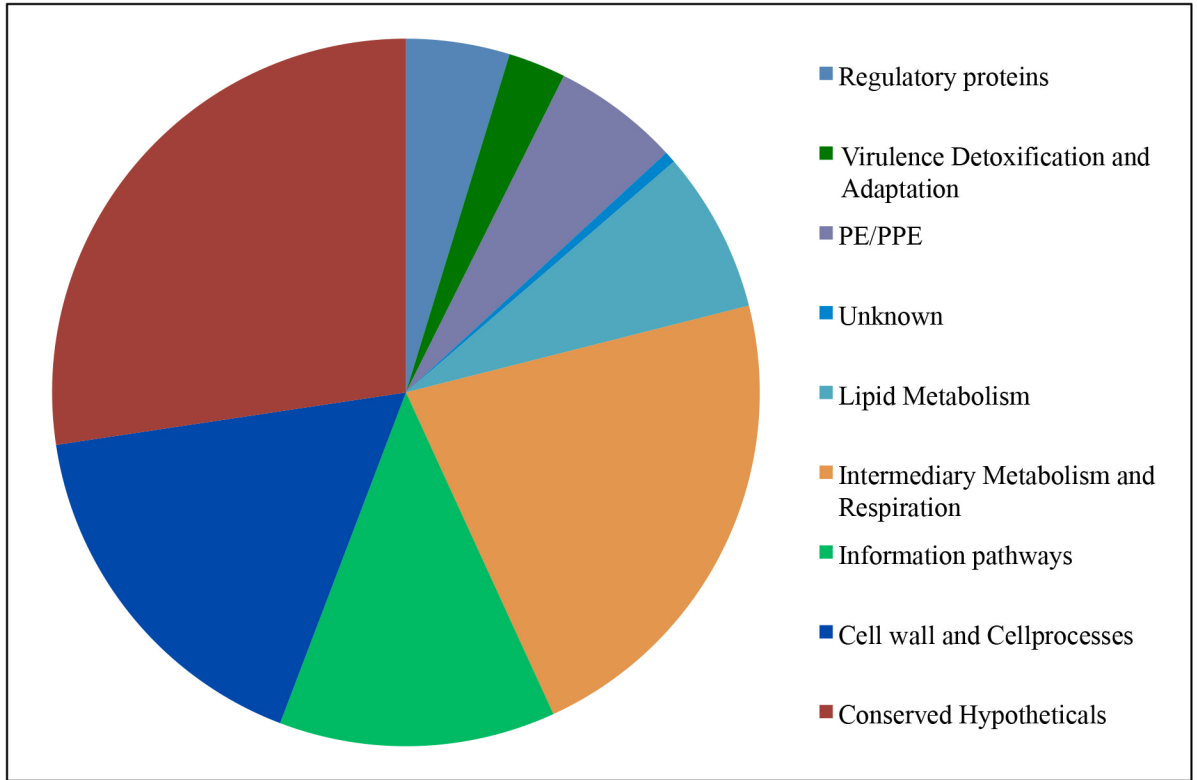

(B)

Gyrase peak distribution

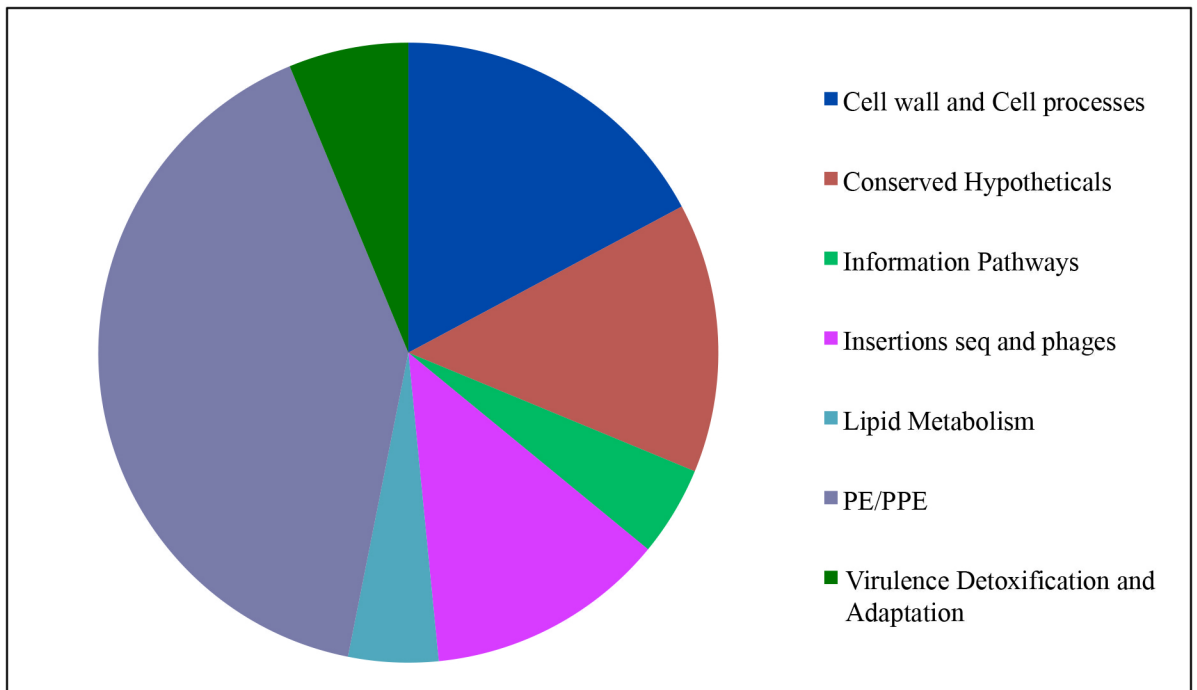

Supplement: S3 Fig — (A) Distribution of Topo I peaks (B) Distribution of gyrase peaks. (PDF) [file pgen.1006754.s003.pdf]

# S5 Fig.

(A)

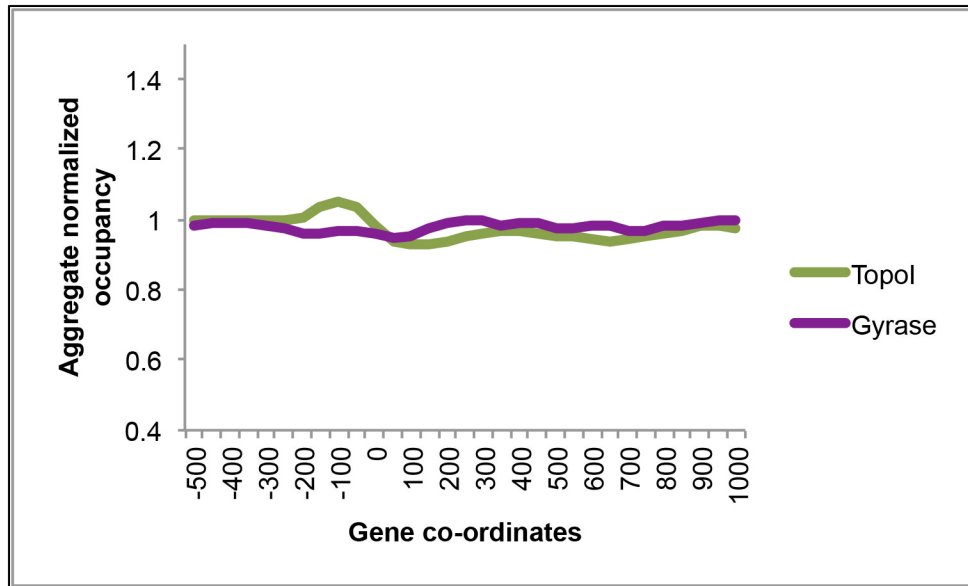

(B)

Topo I and Mock

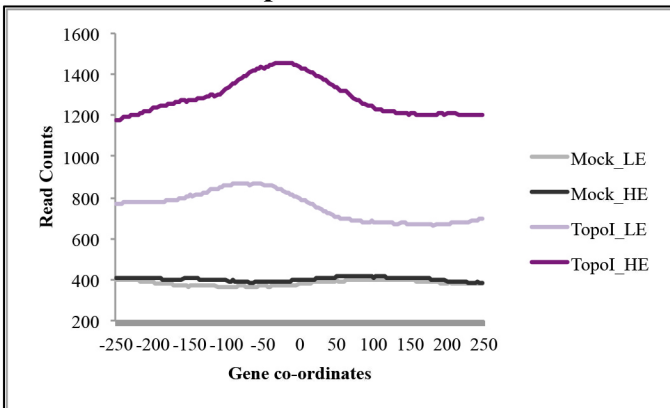

DNA Gyrase and Mock

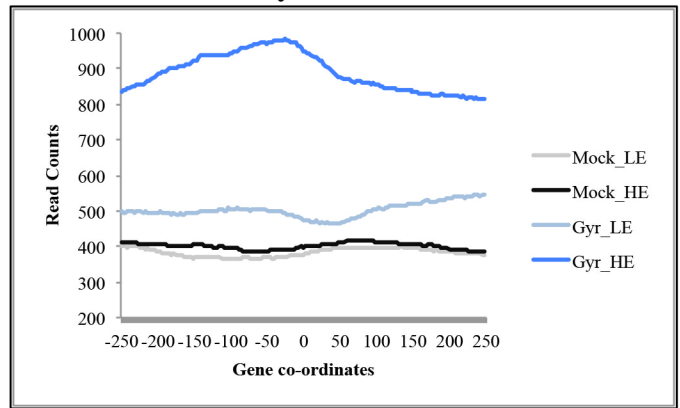

Supplement: S5 Fig — (A) Topo I and gyrase occupancy profile on genes depleted of RNAP. TUs with low transcriptional activity (RPM<1) were selected and analyzed for RNAP enrichment and only TUs with RNAP Enrichment ratio (ER<1) (N = 206) were selected for generating the profile of Topo I and DNA gyrase occupancy around the TSS (0). Mean read counts were calculated in 50 bp sized bins. Data were normalized with the maxima to generate the pattern of distribution on TUs. (B) Evaluation of non-specific ChIP enrichment on highly expressed (HE) genes. The genes were segregated based on RPM values into highly expressed (HE) and low expressed (LE) category as described in Fig 3. The mean read counts were plotted at a single nucleotide resolution around the TSS (-250 to +250) to generate the occupancy profile. (PDF) [file pgen.1006754.s005.pdf]

# S6 Fig.

(A)

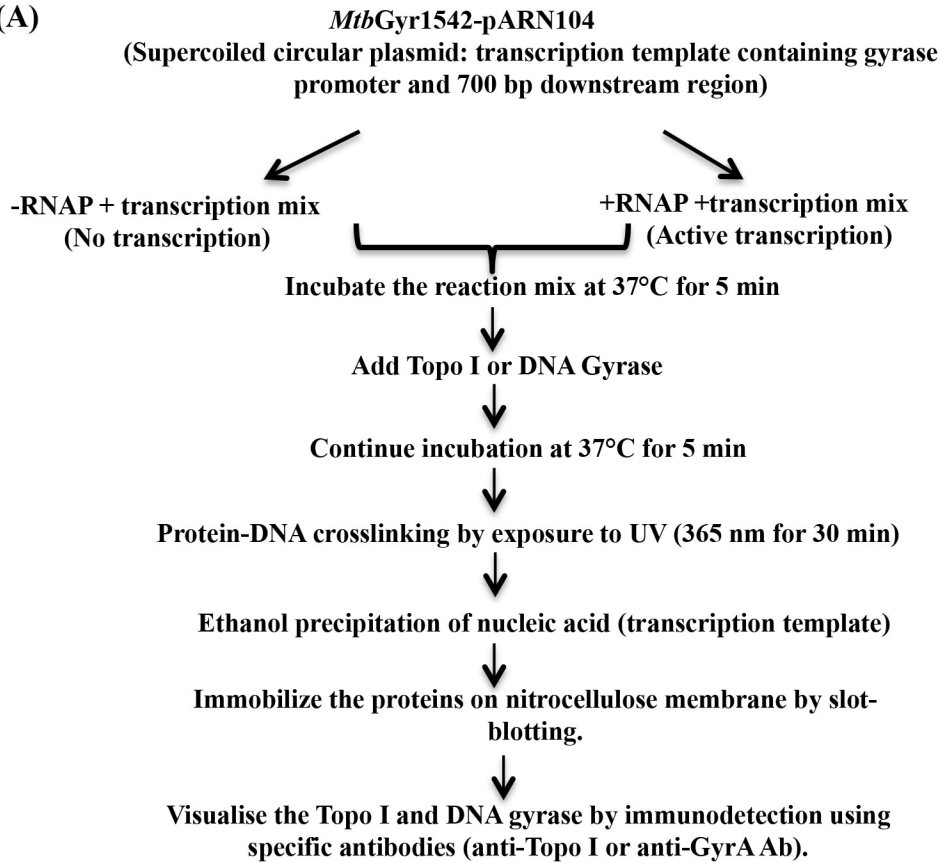

(B)

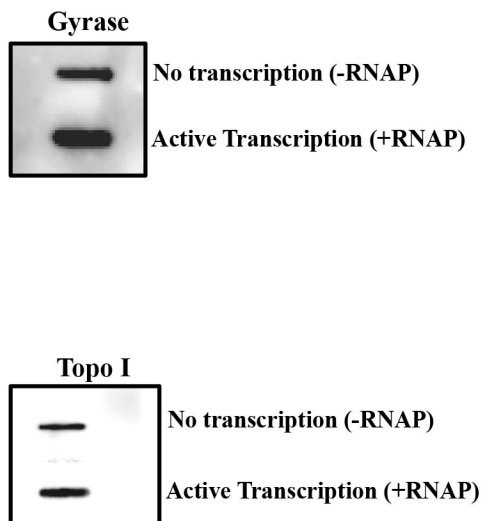

(C)

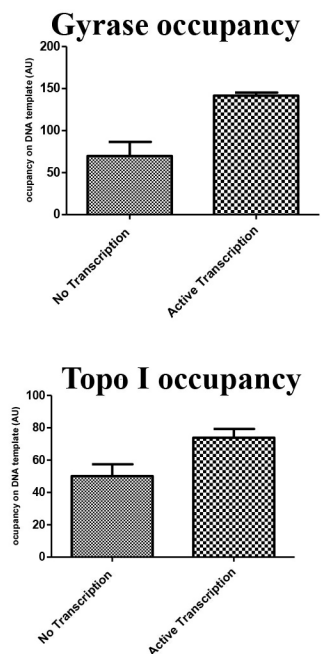

Supplement: S6 Fig — Supercoiled transcription template harboring gyrB promoter and 675 bp transcript coding region were incubated with reaction mix with or without RNAP as depicted in schematic (A). Following the transcription, the accumulated Topo I and DNA gyrase on topologically stressed template were precipitated and detected by immuno-slot blotting (B) and quantification was carried out based on three independent experiments (C). Error bars represent the standard deviation obtained from three independent experiments. (PDF) [file pgen.1006754.s006.pdf]
