# Supplementary material for: Transcription facilitated genome-wide recruitment of topoisomerase I and DNA gyrase
Source: PLoS Genet. 2017 May 2;13(5):e1006754. doi: 10.1371/journal.pgen.1006754 (PMC5433769; doi:10.1371/journal.pgen.1006754)

S4 Fig.

(A) Consensus motifs

Topol recognition motif

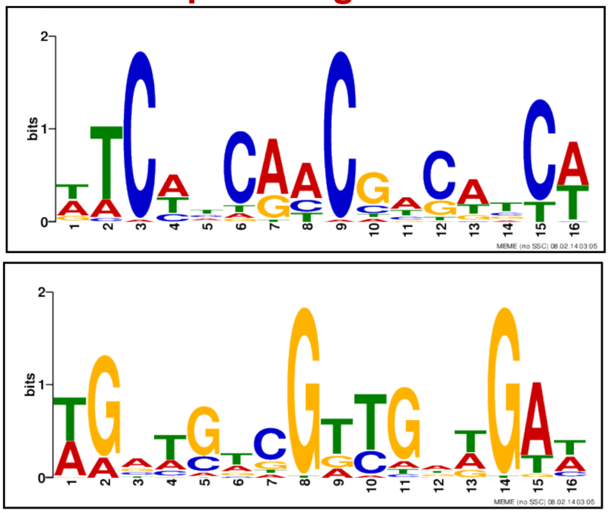

Reverse complement

E-value	9.2e-012
Width	16
Sites	46

Gyrase recognition motif

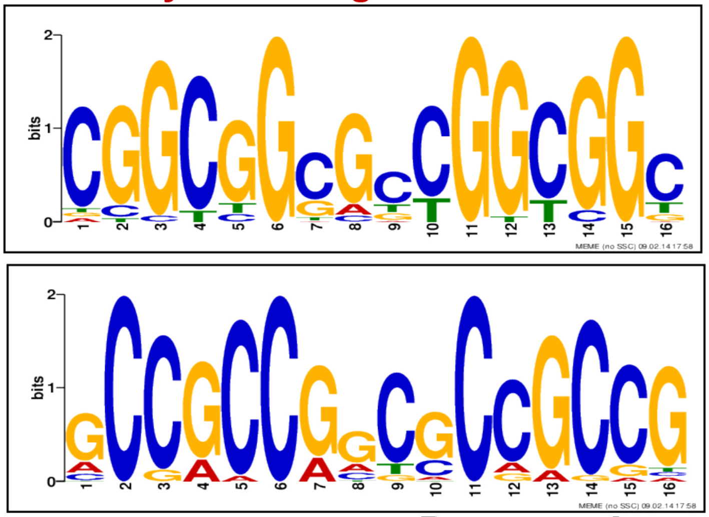

Reverse complement

E-value	8.2e-059
Width	16
Sites	47

(B) Consensus motifs distribution

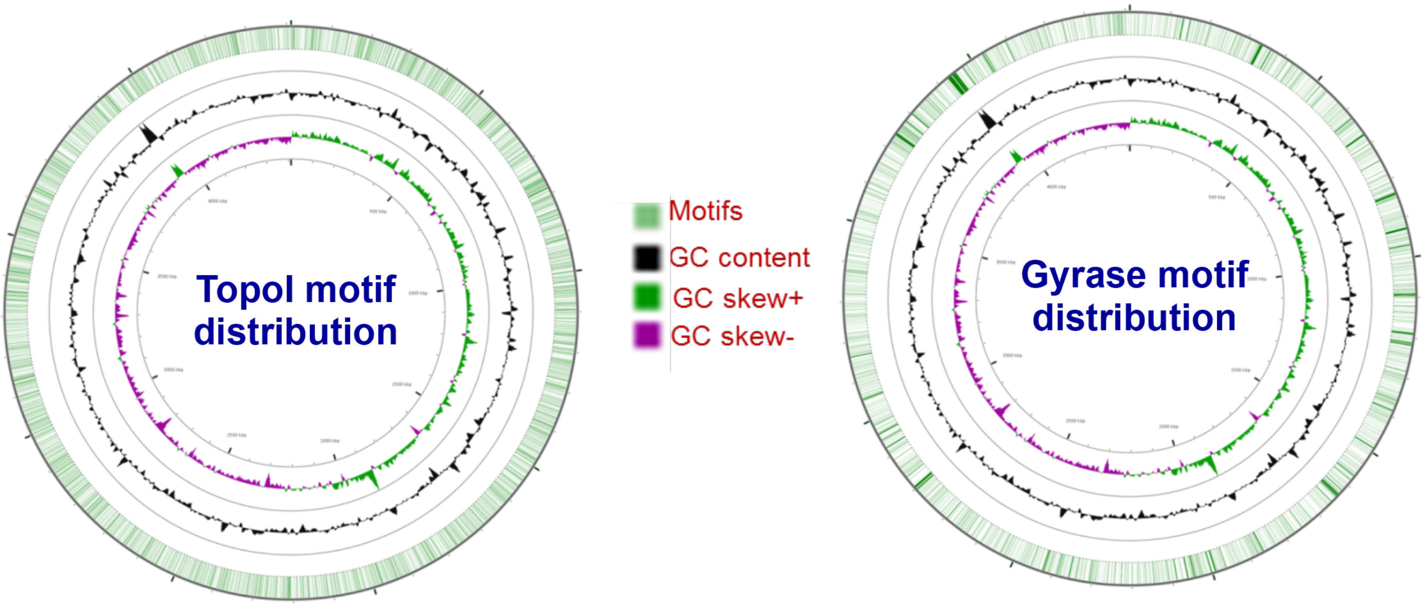

Supplement: S4 Fig — (A) Consensus motifs were obtained from the respective peaks using MEME (B) FIMO detected the genome wide distribution of Topo I and DNA gyrase peaks. The genomic co-ordinates of motifs were taken from FIMO and CG view was used to represent the distribution of consensus motifs on circular genome of Mtb. (PDF) [file pgen.1006754.s004.pdf]
